# The Effect of Needle Reuse on Piglet Skin Puncture Force

**DOI:** 10.3390/vetsci9020090

**Published:** 2022-02-18

**Authors:** Kathryn Owen, Nicola Blackie, Troy John Gibson

**Affiliations:** Department of Pathobiology and Population Sciences, Royal Veterinary College, University of London, Hawkshead Lane, Hatfield AL9 7TA, UK; nblackie@rvc.ac.uk (N.B.); tgibson@rvc.ac.uk (T.J.G.)

**Keywords:** needle reuse, puncture force, scanning electron microscopy, piglet skin, animal welfare

## Abstract

The study investigated whether the repeat use of needles used to inject piglets with iron, influences the force required to puncture into piglet cadaver skin. Pig units (*n* = 31) were surveyed on needle reuse and injection practices, and these findings informed subsequent laboratory-based experiments on needle puncture force into piglet cadaver tissues. A 21 G 5/8” needle was reported as the most used needle type (67.7%), with 80.6% reporting needle reuse; 38.7% changed the needle between litters or earlier if damaged, 16.1% every three litters and 22.5% when it felt blunt or damaged, after each injection session or when changing the bottle of iron solution. There was a significant difference in puncture force between the 1st and 36th use (*p* < 0.05), and between the 1st and 100th use (*p* = 0.0015), but not between the 1st and 12th or 36th use (*p* > 0.999 and *p* = 0.8313, respectively). Scanning electron microscopy (SEM) imaging showed appreciable damage to the needle tip after 12 uses. The repeat use of needles in piglet cadavers increased the force of needle puncture compared to first-time use. When extrapolated to live animals, the use of blunt needles has the potential to cause pain and distress.

## 1. Introduction

There are over 10,000 pig farms in the UK [1] and in 2020, over 8.9 million pigs were reared for slaughter [2]. Indoor pig production accounts for approximately 60% of the UK industry [3]. It is common practice to inject piglets with 200 mg of iron dextran into the ham or neck muscle in the first few days following birth [4]. Parenteral iron administration prevents iron deficiency anaemia, a condition which can reduce growth rates and increase disease susceptibility and mortality [4]. A single dose of iron may not provide sufficient iron for adequate growth, suggesting a requirement for multiple post-natum injections, particularly on organic farms where piglets experience extended suckling periods [5].

The reuse of needles between animals is common in livestock farming. For example, a survey of cattle vaccination practices in the UK found that only 6% (*n* = 4) of respondents used a new needle for each animal [6]. In addition, a survey of sheep farmers (*n* = 360), showed that 3.3% always changed their needle after each animal, 20.3% after 15–20 doses, 32.4% after 50 doses, 39.5% after 100 doses and 4.5% selected the ‘other’ option [7].

Reusing needles to the point of bluntness is contrary to advice from the Agriculture and Horticulture Development Board (AHDB), who state that needles are designed to be used only once, and when injecting a large number of animals should be, at a minimum, changed every ten animals [8]. Food assurance schemes such as Red Tractor also recommend frequent needle changes to reduce injection site lesions and the risks of broken needles contaminating meat products [9]. Iron injection resources offer conflicting advice such as recommending a fresh needle for each piglet [10] or between litters [11]. Automated self-filling syringes encourage needle reuse and are used frequently on intensive-pig farms due to their speed and ease of use. Specialised needles combined with an adaptor to sterilise needles between injections can be fitted. However, needle change is recommended every 100 injections [12] which provides the opportunity for the needle to blunt.

Needle reuse may increase the chance of spreading infection if needles are shared between animals. Otake et al. [13] reported the needle-transmission of porcine reproductive and respiratory syndrome virus to susceptible pigs following intramuscular administration of a Mycoplasma vaccine [13]. Even with subcutaneous injection, reusing the same needle to inject phosphate-buffered saline led to the transmission of bluetongue virus from infected to uninfected ruminants [14]. 

There are limited studies investigating the welfare impacts of the iron administration procedure in piglets; one study showed that injection or oral iron dosing caused similar behavioural and cortisol responses to sham-treated piglets [15]. In humans, the frequency of insertion pain increased with the diameter of the needle [16], and in healthy volunteers, increased in line with the force required to puncture the skin [17]. Others have shown that once a needle has punctured, the tip deforms and tissue products adhere to the damaged metal, which could cause further tissue trauma and pain upon subsequent use [18].

Needles can deform with repeat use and make them susceptible to breakage [19]. Blunt or bent needles could increase tissue damage and cause carcass defects. One study in sows reported lesion prevalence associated with neck and hip injection sites of 11.2% and 2.7%, respectively [20]. Furthermore, the incidence of head and neck abscesses in pigs at slaughter has been estimated at 2.51% over a 3-month period [21]. The number of pigs euthanised due to broken needles that cannot be recovered has been estimated at 1.23 per year per 1200-sow barn, compared to zero reported losses when needle-free systems are used [22]. 

Majcher et al. [23] reported that when punctured into rehydrated leather, single use needles dull and should be considered blunt by the fourth to fifth use. Similarly, the repeat use of 18 G needles in pig cadavers has been shown to blunt the needle after 20 repetitions [19], however damage to the needle tip was not assessed.

The aim of this study was to investigate the current practices and whether the repeat use of needles used to inject piglets with iron, influences the force required to puncture into piglet cadaver skin.

## 2. Materials and Methods

### 2.1. Survey

A survey of UK pig farmers on the current practices of iron injection was conducted between 19 July and 30 September 2019, following approval from the Social Science Research Ethical Review Board of the Royal Veterinary College (SSRERB reference SR 2019-0281). The survey examined farm systems, iron supplementation and injection practices, and needle reuse. See https://rvc.onlinesurveys.ac.uk/copy-survey-of-uk-pig-farmers (accessed on 31 December 2021 for a copy of the survey questions. The survey was disseminated electronically and via post where no email address was available, with the inclusion of a stamped and self-addressed return envelope, to 63 pig farms held on the Animal Husbandry and Extramural Studies database at the Royal Veterinary College. The survey was also sent electronically to the Agriculture and Horticulture Development Board (ADHB), the National Pig Association (NPA), the British Pig Association (BPA) and Scottish Pig Producers (SPP) for further dissemination. 

### 2.2. Laboratory Based Studies

Laboratory studies were conducted following approval from the Clinical Research Ethical Review Board of the Royal Veterinary College (CRERB reference CR2019-M001-2). Needle sharpness was determined by measuring the force required to push the needle through piglet cadaver skin (with sharpness being inversely proportional to the force required) [23]. 

Piglet cadavers were used in four separate experiments: Experiment 1, first-time use (×1) versus blunt needles; Experiments 2 and 3, ×1 versus ×36 needle use; and Experiment 4, comparison of ×1, ×12, ×36 and ×100 needle use. Select needles from Experiments 3 and 4 were analysed by scanning electron microscopy (SEM) (Carl Zeiss GmbH, Oberkochen, Germany). 

Single use, disposable 21 G 5/8” needles (Nutrapet Ltd., East Yorkshire, UK) were used unless otherwise indicated. The blunted 21 G needles used in Experiment 1 were prepared by scraping 10 times against a concrete block, with the bevel pointing towards the operator. The blunted 21 G needles used in Experiments 3 and 4 were prepared by puncturing a chamois leather-covered sponge 17 times. Disposable 18 G 1/2” needles (Agriject/Agrihealth Ltd., Co. Monaghan, UK) were used in Experiments 2 and 4 as controls. 

**Figure 1:**
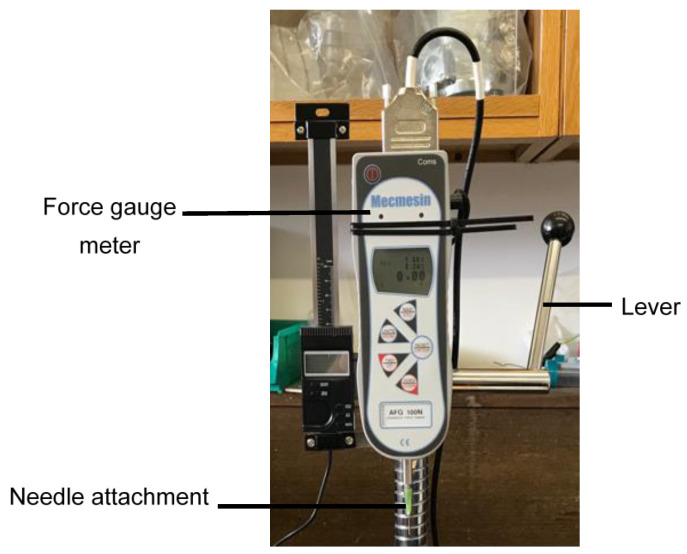
Mecmensin Advanced Force Gauge (AFG 100 N) meter used in puncture studies.

An Advanced Force Gauge (AGF) 100 N digital force gauge meter (Mecmensin Ltd., West Sussex, UK), set at a 10 Hz sampling rate and mounted on a test stand, was used to measure the force of puncture (Figure 1). A gauge extension rod was adapted so that the needles could be attached via the needle hub. Needles were attached with the bevel facing the operator. The gauge meter was set to manual data collection mode and the maximum compression force, measured in Newtons (N), was recorded and then transcribed into Microsoft Excel 2018. 

#### 2.2.1. Piglet Cadaver Studies

**Figure 2:**
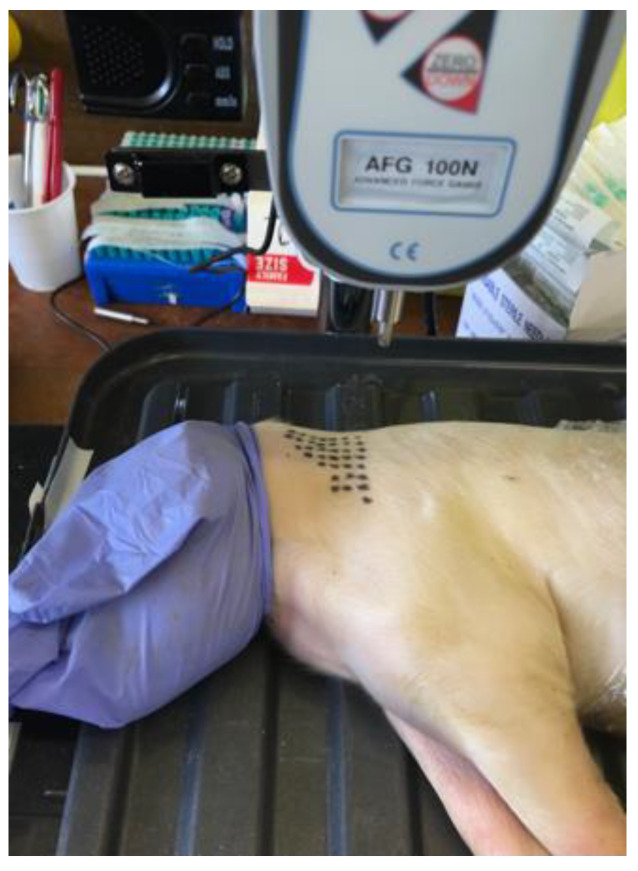
Piglet cadaver positioned with needle attachment directly above the neck injection site (dotted triangle).

Eight piglet cadavers were obtained from a UK pig farm, born alive but following death from natural causes within seven days of birth, and stored at −20 °C until use. The wet weights were between 1.6–2.2 kg (mean 1.9 kg, SD 0.3). The neck region was selected for injection as the results of the survey showed that 18/31 (58%) of the respondents injected into the neck. A triangle was drawn on one side of the neck behind the ear, with 35 evenly spaced dots, each one being a potential puncture site (Figure 2), as this is where the injection would normally occur [4]. The piglet cadavers were secured to prevent lateral movement and positioned so that when the lever was lowered, the needle punctured the neck, stopping at the shaft just prior to the plastic hub. The needles were punctured into the cadaver at 90 degrees and over approximately 2 s, repositioning to a randomised new site using Research Randomizer (https://www.randomizer.org/ (accessed on 31 December 2021)). 

The repeat use 21 G needles were prepared by injecting the needles randomly into the muscle of the piglet cadaver using an automated injector gun (1 mL, Nutrapet Ltd., East Yorkshire, UK), changing the needle every 10 punctures so that any effect of cadaver drying whilst the needles were being prepared, would be spread across all groups. The designs of Experiments 1–4 were as follows:

- Experiment 1: First-time use (×1) needles (*n* = 18) versus blunt needles (*n* = 17), prepared by scraping 10 times on concrete. Single punctures into the neck alternated between ×1 and blunt needles. One piglet cadaver was used.
- Experiment 2: First-time use (×1) versus ×36 needle use. Puncture 1 into the neck of a piglet cadaver (*n* = 10) (×1A). The needles were then injected randomly 34 times across the remaining cadaver with the 36th puncture into the neck (*n* = 10) (×36A). To assess whether the piglet cadaver dried whilst the ×36A needles were being prepared, the puncture of the ×36A needles were alternated with a new set of needles (×1B) (*n* = 10). The 18 G ½” needles (*n* = 5) were used as a positive control (18 G) as puncture force has previously been shown to increase with needle diameter [24]. One piglet cadaver was used.
- Experiment 3: The design was the same as for Experiment 2 except that for each test the group *n* = 9, and the needles previously blunted by puncture into chamois leather 17 times (*n* = 6), were used as a positive control. One piglet cadaver was used.
- Experiment 4: Comparison of ×1, ×12, ×36 and ×100 punctures. The repeat use needles were prepared by injecting randomly 11, 35 or 99 times (across four piglet cadavers), *n* = 7 per group. The 18 G ½” needles (18 G C) (*n* = 3) and the needles previously blunted by puncture into chamois leather 17 times (21 G C) (*n* = 6) were used as positive controls. The order that the needles were punctured into the neck of a fifth piglet cadaver were randomised using Research Randomizer. Five piglet cadavers were used in total.

#### 2.2.2. SEM Analysis

**Figure 3:**
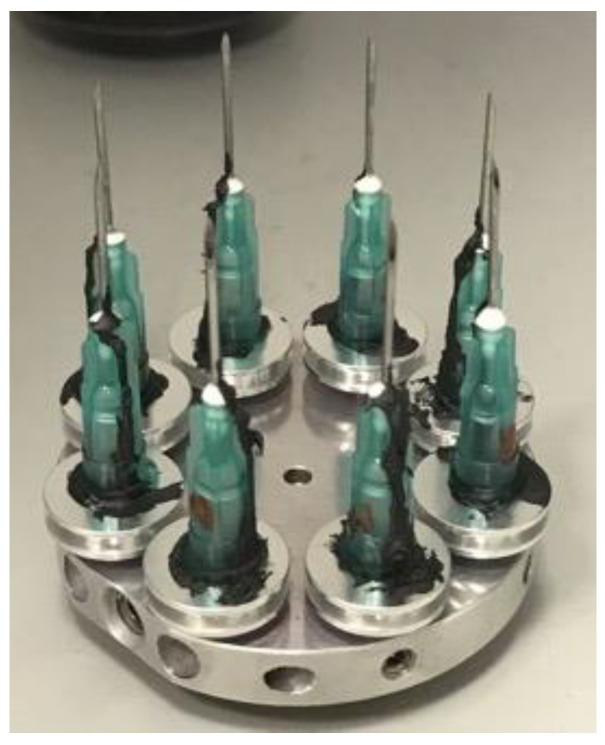
Needles mounted on aluminium specimen stubs with Leit-C to form a conducting strip from the base of the hub to the needle shaft.

Following the cadaver studies, needles from Experiments 3 and 4 and the unused control needles were soaked in 1% Virkon disinfectant (Antec International Ltd., Suffolk, UK) for 30 min and rinsed in water. They were then air-dried and dipped in acetone in preparation for SEM. Two needles per data point were analysed and the needles with puncture forces closest to the median value of each run were selected. The needles were mounted on 0.5” aluminium specimen stubs (Agar Scientific Ltd., Essex, UK) with Leit-C for microscopy (Fluka Analytical/Sigma Aldrich, Dorset, UK) from the base of the hub to the needle shaft to form a conducting strip (Figure 3). Images were captured using a Zeiss Supra 40 scanning electron microscope (Carl Zeiss GmbH, Oberkochen, Germany) with 10 kV accelerating voltage in a high current mode. 

## 3. Results

### 3.1. Survey

There were 36 replies to the survey, of which two responses did not complete the survey, two farms contacted were no longer in business and one respondent made an on-line entry but no response to the questions. Consequently, there were 31 material responses (20 online and 11 postal), as summarized in Table 1. The iron injection of piglets was reported in 28/31 (90.3%) responses, of which one response reported the use of a needle-free injection system which uses pressure to penetrate the iron solution through the skin and into the muscle; 2/31 (6.5%) reported no iron supplementation; and 1/31 (3.2%) administered an oral iron solution. The most common site of injection was the neck (18/31; 58.1%). The 21 G 5/8” needle was the most common needle type used for iron injections (21/31; 67.7%). Needle reuse was reported in 25/31 (80.6%) responses, with only 38.7% (*n* = 12) changing the needle between litters (10–12 piglets), or earlier if damaged, and 16.1% (*n* = 5) changing every three litters (approx. 36 piglets). Twenty-two and a half percent (*n* = 7) reported only changing the needle when it felt blunt or damaged, after each injection session or when changing the bottle of iron solution. 

### 3.2. Piglet Cadaver Studies

**Figure 4:**
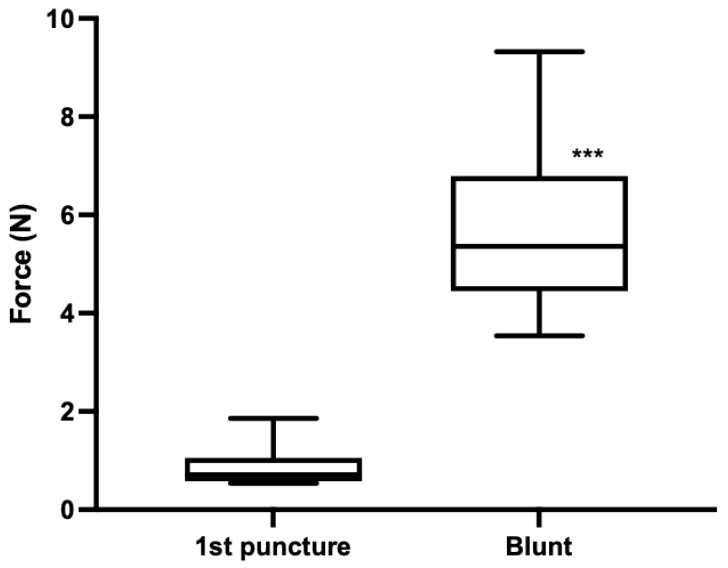
Experiment 1. Puncture force (N) into piglet cadaver for 1st puncture (*n* = 18) and artificially blunted (*n* = 17) 21 G needles. *** *p* < 0.0001 compared to 1st puncture.

Experiment 1: The force required to puncture piglet skin with 21 G needles ranged from 0.54 to 1.86 N, with a median of 0.71 N (*n* = 18) (Figure 4). The needles that had been purposely blunted produced a puncture force ranging from 3.54 to 9.32 N, with a median of 5.36 N (*n* = 17). There was a significant difference in the force of puncture with unused needles compared to needles that were visibly blunt (*p* < 0.0001). 

**Figure 5:**
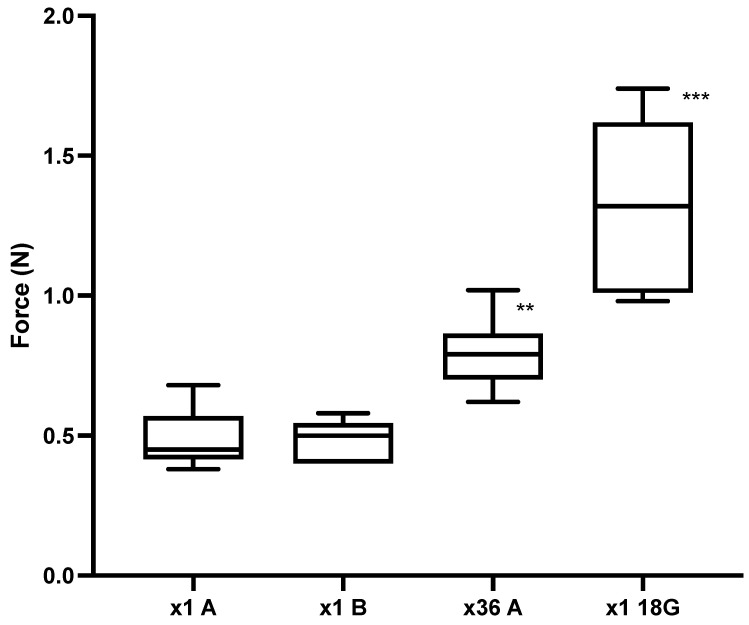
Experiment 2. Puncture force (N) into piglet cadaver for ×1A, ×1B and ×36A 21 G needles and ×1 18 G control needles (*n* = 10 needles per group). ** *p* = 0.0023 compared to ×1A, *** *p* < 0.0002 compared to ×1A.

Experiment 2: Figure 5 shows that the force required for needle A to initially puncture the piglet skin ranged from 0.38 to 0.68 N, with a median of 0.45 N (*n* = 10) (×1A), which increased to 0.79 N (range 0.62–1.02 N, *n* = 10) upon the 36th puncture (×36A). The force required for ×1B needles to initially puncture the piglet skin ranged from 0.4 to 0.58 N, with a median of 0.50 N (*n* = 10). There was a significant difference in the puncture force between ×1A compared to ×36A (*p* = 0.0023), but there was no significant difference between ×1A and ×1B (*p* > 0.999). The 18 G needles were used as a positive control and the median puncture force was 1.32 N (range 0.98–1.74 N, *n* = 10). This was significantly different to ×1A (*p* < 0.0002). 

**Figure 6:**
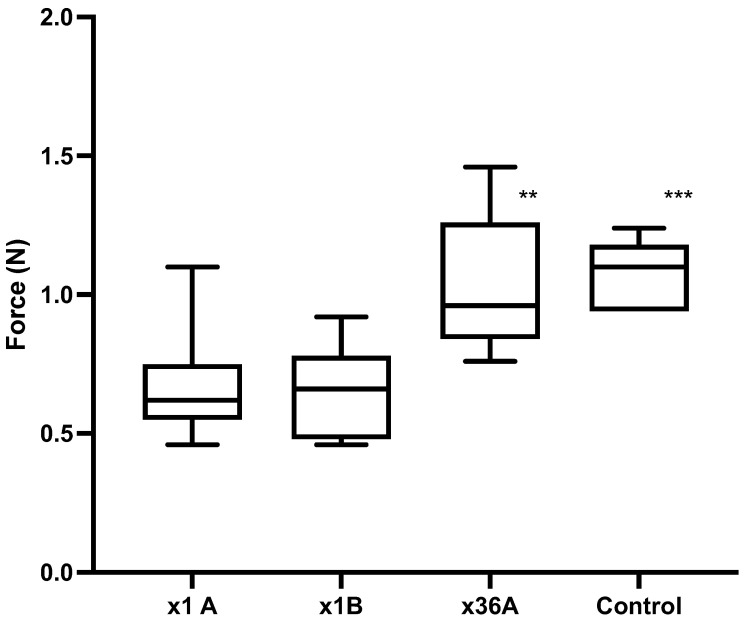
Experiment 3. Puncture force (N) into piglet cadaver for ×1A, ×1B and ×36A (*n* = 9 per group) and control 21 G needles (blunted by puncture into chamois leather) (*n* = 6). ** *p* = 0.0111 compared to ×1A, *** *p* = 0.0053 compared to ×1A.

Experiment 3: The median force for ×1A, ×1B and ×36A were 0.62 N (range 0.46–1.10 N, *n* = 9), 0.66 N (range 0.46–0.92 N, *n* = 9), and 0.96 N (range 0.76–1.46 N, *n* = 9), respectively (Figure 6). There was a significant difference in the puncture force between ×1A compared to ×36A (*p* = 0.0111), but not between ×1A and ×1B (*p* > 0.9999). The 21 G needles blunted by puncture into chamois leather were used as a positive control (Control) and gave a median force of 1.10 N (range 0.94–1.24 N, *n* = 6). There was a significant difference between the Control and the ×1A groups (*p* = 0.0053).

**Figure 7:**
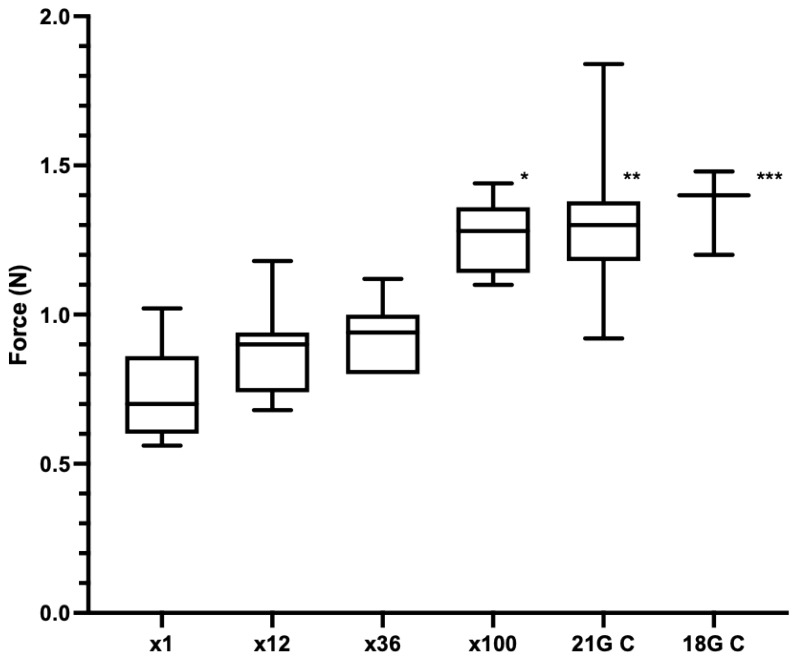
Experiment 4. Puncture force (N) into piglet cadaver for 1st, 12th, 36th or 100th puncture of 21 G needles (*n* = 7 per group) and control 21 G needles (blunted by puncture into chamois leather) (21 G C), (*n* = 6) and control 18 G needles (18 G C), (*n* = 3). * *p* = 0.0015 compared to ×1, ** *p* = 0.0017 compared to ×1, *** *p* = 0.0041 compared to ×1.

Experiment 4: The median forces of the punctures for ×1, ×12 and ×36 were 0.70 N (range 0.56–1.02 N, *n* = 7), 0.90 N (range 0.68–1.18 N, *n* = 7) and 0.94 N (range 0.80–1.12 N, *n* = 7), respectively (Figure 7). There was no significant difference between 12 or 36 injections compared to the first injection (*p* > 0.999 and *p* = 0.8313, respectively). The median force at 100 injections was 1.28 N (range 1.10–1.44, *n* = 7), which was significantly different to the first puncture (*p* = 0.0015). The 21 G needles blunted by puncture into chamois leather (21 G C) and the 18 G needles (18 G C) were used as positive controls. The median force of 21 G C was 1.30 N (range 0.92–1.84 N, *n* = 7) and of 18 G C was 1.40 N (range 1.20–1.48 N, *n* = 3), which were both significantly increased compared to first-time needle use (*p* = 0.0017 and *p* = 0.0041, respectively). 

#### Variation across Piglet Cadavers 

The median force of the first puncture across Experiments 1–4 was 0.60 N (range 0.38–1.86 N, *n* = 63). There was a significant difference in the first puncture force between Experiments 1 and 2 (*p* < 0.0001), but not between Experiments 1 and 3 (*p* = 0.4392) or Experiments 1 and 4 (*p* > 0.9999). 

### 3.3. SEM Analysis

**Figure 8:**
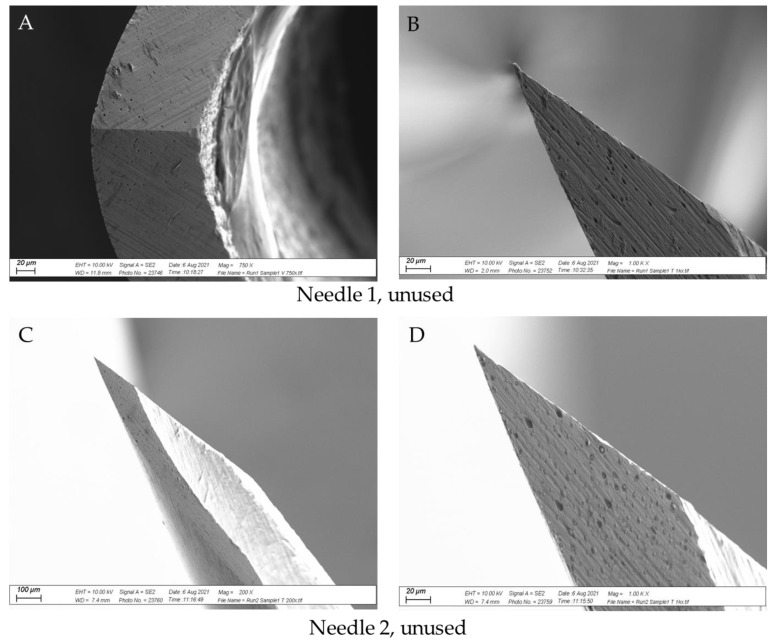
SEM images of two unused 21 G needles (Needle 1 and Needle 2). Image (**A**): Needle 1 (magnified ×750), Image (**B**): Needle 1 (magnified ×1000), Image (**C**): Needle 2 (magnified ×200), Image (**D**): Needle 2 (magnified ×1000).

**Figure 9:**
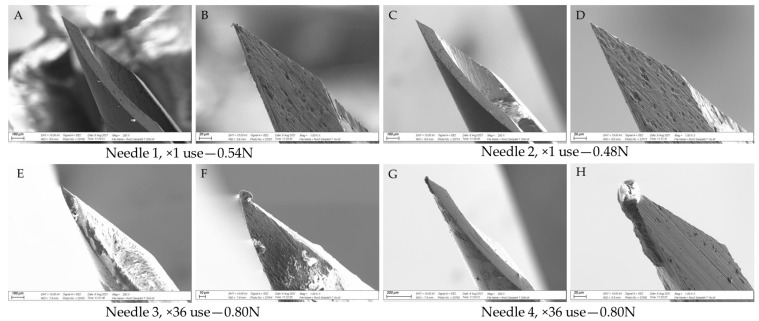
SEM images of select needles from Experiment 3 together with the puncture force (N) measured upon injection into a piglet cadaver tissue. Needle 1, ×1 use, puncture force 0.54 N; Needle 2, ×1 use, puncture force 0.48 N; Needle 3, ×36 use, puncture force 0.80 N; Needle 4, ×36 use, puncture force 0.80 N. Image (**A**): Needle 1, ×1 use (magnified ×200); Image (**B**): Needle 1, ×1 use (magnified ×1000); Image (**C**): Needle 2, ×1 use (magnified ×200); Image (**D**);: Needle 2, ×1 use (magnified ×1000); Image (**E**): Needle 3, ×36 use (magnified ×200); Image (**F**): Needle 3, ×36 use (magnified ×1000); Image (**G**): Needle 4, ×36 use (magnified ×200); Image (**H**): Needle 4, ×36 use (magnified ×1000).

**Figure 10:**
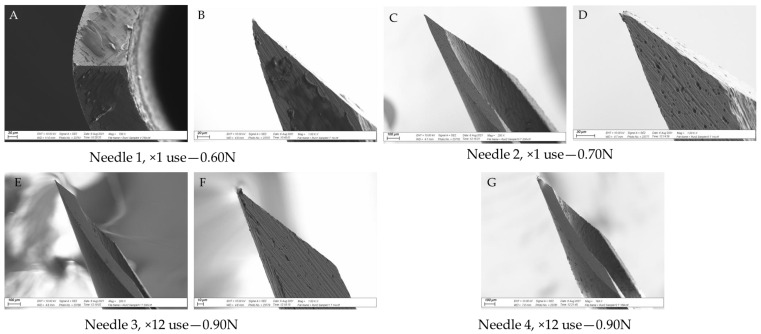

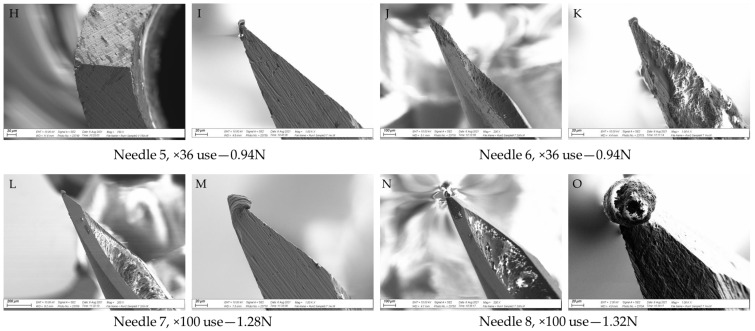
SEM images of select needles from Experiment 4 together with the puncture force (N) measured upon injection into a piglet cadaver tissue. Needle 1, ×1 use, puncture force 0.60 N; Needle 2, ×1 use, puncture force 0.70 N; Needle 3, ×12 use, puncture force 0.90 N; Needle 4, ×12 use, puncture force 0.90 N; Needle 5, ×36 use, puncture force 0.94 N; Needle 6, ×36 use, puncture force 0.94 N; Needle 7, ×100 use, puncture force 1.28 N; Needle 8, ×100 use, puncture force 1.32 N. Image (**A**): Needle 1, ×1 use (magnified ×750); Image (**B**): Needle 1, ×1 use (magnified ×1000); Image (**C**): Needle 2, ×1 use (magnified ×200); Image (**D**): Needle 2, ×1 use (magnified ×1000); Image (**E**): Needle 3, ×12 use (magnified ×200); Image (**F**): Needle 3, ×12 use (magnified ×1000); Image (**G**): Needle 4, ×12 use (magnified ×169); Image (**H**): Needle 5, ×36 use (magnified ×750); Image (**I**): Needle 5, ×36 use (magnified ×1000); Image (**J**): Needle 6, ×36 use (magnified ×200); Image (**K**): Needle 5, ×36 use (magnified ×1000); Image (**L**): Needle 7, ×100 use (magnified ×200); Image (**M**): Needle 7, ×100 use (magnified ×1000); Image (**N**): Needle 8, ×100 use (magnified ×200); Image (**O**): Needle 8, ×100 use (magnified ×1000).

Figure 8 shows SEM images of two unused needles, Figure 9 shows images from two ×1 use needles and two ×36 use needles from Experiment 3, and Figure 10 shows images from two needles per data point from Experiment 4. Together, the images show that the tip of the needle became damaged with repeated use, in line with the increase in puncture force. Appreciable damage was detected after 12 uses, with some evidence that the needle was altered after a single use (Figure 9B and Figure 10A,B,D). The most significant damage was noted after 100 uses (Figure 10L–N,O). Residual tissue products were present on the bevel surface of several needles after one and 36 uses (Figure 9C,E,F and Figure 10A,J,K). There was also evidence of residual tissue on the bevel and tip of needles after 100 uses (Figure 10L,N,O).

## 4. Discussion

### 4.1. Survey

Although a small sample size, all the respondents to the survey reported needle reuse between piglets. Furthermore, a number of pig units reported only changing the needle when it is visibly blunt, damaged or when changing the bottle of iron, which could be following as many as 250 injections. The finding that the 21 G 5/8” needle was the most common needle type used for iron injections informed the subsequent piglet skin cadaver experiments. 

### 4.2. Repeated Needle Use

The data show that the repeated use of a 21 G needle resulted in a blunter needle, with more force required to puncture the piglet skin compared to first-time use. There was a significant difference in the puncture force on the 36th injection in two out of three experiments, which indicated that the needle could blunt when changing the needle every three litters. Even assuming the needle did not blunt significantly until its 100th use, reusing a needle 100 times in one session is not unrealistic given that this equates to approximately ten piglet litters. 

There was no difference in the force by the 12th puncture, which contrasts to other studies where single use needles (14–20 G) dulled within two to three uses and were considered blunt by the fourth to fifth use [23]. However, these experiments used rehydrated leather hide which was not representative of skin layers and differences in the moisture content across the leather may have biased the results [23]. The force of the first puncture was more than 20 N [23], which is more than thirty times the puncture force measured with 21 G needles in this study. Previous work has shown that puncture force increased with needle diameter [24] and so a lower puncture force using 21 G needles would be expected. However, diameter alone is unlikely to account for the large difference observed between the two studies and suggests that the specific properties of the puncture material may influence the force of puncture and subsequent rate of needle blunting, rather than the number of times the needle is used per se. One possibility is that there is an inverse relationship between initial puncture force and the number of repeat punctures required to blunt the needle. This could be investigated by correlating initial puncture force with the number of repeat punctures required for the needle to blunt, across a range of material types. 

When 18 G needles were used as a positive control in this study, they produced a median puncture force of 1.32 N, which is comparable to the initial puncture force of 1.8 N in the neck region of pig cadavers reported elsewhere [19]. In that study, puncture force increased to 4.4 N after 10 repetitions, which plateaued to 5.8 N after 20 repetitions [19], an approximate 3-fold change from first-time use. Similarly, others [23] reported an approximately 3-fold increase in force from initial puncture by the time a needle was maximally blunt. In this study, a 1.9-fold increase from the initial puncture force of a 21 G needle was observed by the 100th use. Further repetitions beyond 100 injections would determine whether the needle was maximally blunt by the 100th use or if there was further scope for the needle to blunt. Experiment 1 showed that purposely blunted needles produced a 7.5-fold increase from the initial puncture force, but this is less relevant to the on-farm setting since they were artificially produced.

Measuring the force of needle puncture is a measurement of needle efficiency, with needle blunting a measurement of reduced needle efficiency. The use of blunt needles for the iron injection of piglets has the potential for piglets to experience pain and distress, which may lead to a significant welfare compromise. However, given that needle blunting is a proxy for increased tissue damage, and this is in itself a proxy for increased pain, robust studies to determine whether piglets experience pain or stress due to the increased puncture force observed within this study are needed. Previous studies comparing iron injection to oral administration failed to distinguish between handling stress and stress due to the route of iron administration [15]. Furthermore, needle-free intradermal injection reduced aversive behaviours in piglets compared to intramuscular injection [25,26]. However, volume differences may account for the increased inflammatory response seen via the intramuscular route, rather than the force of the needle tip itself. Nonetheless, the ability to distinguish between a sharp or blunt stimulus has been shown to depend on A-delta sensory nerve fibre function [27] and it is reasonable to suggest that using a damaged needle would modulate this response.

There was no difference in the initial puncture force between the piglets used in Experiments 1, 3 and 4, indicating reproducibility in three out of four cases. However, the initial puncture force achieved in Experiment 2 was significantly less than that of Experiment 1. The lower force could be due to smaller size or damage to the piglet skin which made it easier to puncture. Potential variation across piglets was considered in the design of this study, as repeat use needles were prepared by random puncture across cadavers, to ensure any variation was evenly spread across all groups. Other work has shown there to be variability in the puncture force across different regions of the piglet hide, with the neck region producing the lowest puncture force [19]. This raises the question of whether it was appropriate to use the entire cadaver to prepare the repeat use needles. However, the study was limited by the number of piglets available and at least using an automated gun replicated the injection method commonly used on farms. 

### 4.3. SEM Assessment of Needle Tip Damage

The results support the hypothesis that needle damage would be detected by SEM, with increased damage to the needle tip as the puncture force increased. Damage to the needle tip upon one-time use has been previously reported [18], although this may relate to the properties of the puncture material, not necessarily the frequency of use. Tissue products adhered to several needle tips, and this may represent a higher disease transmission risk, as there is an opportunity to transfer iatrogenic material from one animal to the next. In this regard, Ko et al. [28] noted that injection site lesions in pigs vaccinated via transdermal needle-free routes were 14.8% lower than pigs vaccinated using a conventional needle. However, four times less vaccine was administered transdermally, likely contributing to a greater inflammatory reaction in the needle group [28]. King et al. [21] noted significantly more abscesses at slaughter in pigs on the right side of the neck than on the left, and speculated that piglets were likely injected with iron on the right by a lone operative, due to most people being right-handed. If so, the repeat use of needles amongst piglets could contribute to tissue damage and increase the likelihood of abscess development. Furthermore, the greater force required to inject piglets when using a damaged needle may contribute to needle stick injuries in swine-operatives, where rates of 2.13% per 100,000 full time equivalents per year have been reported [22].

### 4.4. Study Design

In this study, efforts to ensure that experiments were randomised appropriately were taken. This included randomising the order of puncture, the specific puncture site within the neck and the preparation of the repeat use needles across the cadavers. Furthermore, there was no difference between ×1A or ×1B in Experiments 2 and 3, indicating that there was no appreciable drying of the cadaver during the time it took to prepare the ×36 use needles.

There was variation between experiments in the force required to puncture the skin after 36 uses. Experiments 2 and 3 demonstrated that by 36 uses, significantly more force was required. However, Experiment 4 found no difference by 36 punctures compared to first-time use. It must be noted that Experiment 4 used fewer replicates than the other experiments, thus, reducing statistical power. In addition, due to the low numbers of piglet cadavers collected, puncture force was only measured at the 1st, 12th, 36th and 100th puncture, whereas ideally, consecutive measurements would have been taken to more precisely assess when the needle blunts. However, despite these limitations, this study demonstrates that significantly more puncture force was required when the needles were reused. 

This study is limited in that it was not practical to use fresh piglet cadavers and the piglets were frozen prior to use. The freeze-thaw cycle could affect the force of needle puncture. However, a median puncture force of 0.60 N was measured in this study. This is similar to earlier work [19] which reported an initial puncture force of 0.66 N from fresh pig cadavers, although they did not specify the age of the cadaver and also used a wider diameter 20 G needle.

Needles from only one manufacturer were assessed in this study. Follow up work should expand the range of needles tested as there could be differences in performance between brands. Another area for future study will be to collect needles disposed from pig farms for imaging and to measure the puncture force, in relation to the needle brand and the operator technique, so that on-farm conditions can be assessed. Further studies to assess whether using a blunter needle heightens pain or a stress response in vivo will be needed to further understand the welfare implications of needle reuse.

## 5. Conclusions

In summary, this study has shown that repeated use of a 21 G needle, the most common size of needle used to inject piglets with iron, increased the force of needle puncture in piglet cadavers, compared to a single use. A difference in force could be detected by 36 uses, although SEM imaging detected damage to the needle tip by 12 uses. The data highlights the potential importance of changing the needle frequently, and certainly within three litters or less, to mitigate against the potential to cause pain and stress. This study should not be considered to provide data to support the reuse of needles, but rather to demonstrate that needles become blunt and damaged with repeated use. 

## Figures and Tables

**Table 1:** Summary of the results of the pig unit survey on needle reuse and injection practices.

Parameter	Category	Frequency	Percentage
Was iron supplementation of piglets conducted? (*n* = 31)	Yes (Injection)	28	90.3%
	Yes (Oral)	1	3.2%
	No	2	6.5%
Injection Site (*n* = 31)	Neck	18	58.1%
	Leg	9	29.0%
	Neck or Leg	1	3.2%
	Not applicable	3 *	9.7%
Volume of iron dextran injected ** (*n* = 30)	1 mL 200 mg/mL	26	86.7%
	2 mL 100 mg/mL	1	3.3%
	Not applicable	3 *	10.0%
Use of automated injector ** (*n* = 20)	Yes	17	85.0%
	No	3	15.0%
Size of needle (*n* = 31)	21 G 5/8”	21	67.7%
	21 G 1/2”	1	3.2%
	21 G 3/8”	1	3.2%
	19 G 1”	1	3.2%
	20 G 3/8”	1	3.2%
	23 G 1/1/4”	1	3.2%
	No injection	4 *	12.9%
	Unclear	1 *	3.2%
Frequency of needle change (*n* = 31)	Every litter (10–12 piglets)	8	25.8%
	Every litter or earlier if blunt	4	12.9%
	Every other litter	1	3.2%
	Every 3 litters	5	16.1%
	When blunt/changing bottle/after injection session/damaged	7	22.6%
	Not applicable	4 *	12.9%
	Unclear	2 *	6.5%

* Two respondents reported no iron administration, one reported the use of a needle-free injection system, one reported oral iron administration and other responses were considered unreliable/unclear. ** Question was not answered by some respondents.

## Data Availability

The data presented in this study are available on request from the corresponding author.
